# 

**Published:** 1984-04

**Authors:** 


APRIL 1984

VOLUME 99 (ii)

NUMBER 370

Published Quarterly

Annual Subscription £8 Post Free

### BRISTOL MEDICO-CHIRURGICAL JOURNAL

Contents

39. **Editorial**

Established 1883

### BRISTOL MEDICO-CHIRURGICAL JOURNAL

Incorporating the Medical Journal of the South West

**74.** **Book Review**

**75.** **Correspondence**

**75.** **Examination Results**

Printed in Great Britain by John Wright & Sons (Printing) Ltd, at The Stonebridge Press, Bristol

### Editorial Committee

EDITOR
*Mr. M. G. Wilson*
*Litfield House, Clifton, Bristol BS8 3LS*
MEMBERS
*Dr. M. B. Lennard*
*Dr. D. W. Wright*
*The Hon. Treasurer of the Society*
*The Hon. Secretary of the Society*
SECRETARY
*Mr. B. P. Jones, The Library, Medical School, University Walk, Bristol BS8 1TD*

### Correspondents

*Dr. J. D. Davies*
*Dr. J. Jancar*
*Dr. H. G. Mather*
*Professor G. M. Stirrat*
*Dr. J. L. G. Thomson*
*Dr. M. J. Whitfield*
*Professor R. C. N. Williamson*
*Dr. D. W. Wright*

### Bristol Medico-Chirurgical Society

PRESIDENT 1983/84
*Dr. N. C. Tricks*
PRESIDENT ELECT
*Dr I. S. Bailey*
HONORARY SECRETARY
*Dr. H. M. Norman*
*Hillside, Vicarage Lane, Barrow Gurney*
HONORARY TREASURER
*Dr. J. B. Penry.*
*Southmead Hospital, Bristol BS10 5NB*

The Society was founded in 1874. In 1883 publication of the Journal began and has continued quarterly ever since then. During the years 1949 to 1962 it appeared under the title of 'The Medical Journal of the South West'.

### Notice to Contributors

*The Journal is especially concerned to provide a place for the recording of medical thought, interests and practice in and around Bristol. It therefore welcomes articles that, as well as being of immediate interest to members, will document the local and contemporary medical scene for our successors.*

*Original articles are preferred in the form proposed by the International Committee of Medical Editors (The Vancouver System) and published as an article in the British Medical Journal in 1979 (Br. Med. J.* **1***, 532–535). This has been reprinted as a pamphlet and may be obtained from the Editor of the B.M.J., B.M.A. House, Tavistock Square, London WC1H 9JR (enclose 50p).*

*Also welcome are letters to the Editor, Book Reviews, news of important appointments and events, etc.*

### UNIVERSITY OF BRISTOL FACULTY OF MEDICINE FINAL EXAMINATION FOR THE DEGREES OF M.B., Ch.B.

**December 1983**

Harnam Singh AULAKH
Diane Marie BUNGAY
Hilary Krystyna FAREY – with Distinction in Obstetrics and Gynaecology
Stephen Mark FRANKLIN
Garry Wayne HENRY
Martyn Frank HEWETT
Alistair Malcolm Westwood HOLMES
Christopher Michael Periam MATHER
Stephen William PEARSON
Joan PLATFORD

### THE DEGREE OF MASTER OF SCIENCE IN MEDICAL SCIENCES

**January 1984**

Lesley Giller JONES

**March 1984**

Anna Maria PALMER

### THE DEGREE OF DOCTOR OF PHILOSOPHY

**January 1984**

Zuhair Ahmad Mohammad AL-CHALABY
Ian David BROWN
David John HAMPSON
Raymond Henry POULDING
Karin STEVENS

### THE DEGREE OF DOCTOR OF MEDICINE

**January 1984**

Andrew Justin BALL
Robin Paul BOLTON
Kevin Michael DE COCK
Mokelu Dan ENE
Susan Margaret TUCK

**March 1984**

Barry John FAIRBROTHER
Paul John FINAN

